# Inter-Laboratory Reproducibility of Inducible HIV-1 Reservoir Quantification by TILDA

**DOI:** 10.3390/v12090973

**Published:** 2020-09-02

**Authors:** Cynthia Lungu, Francesco A. Procopio, Ronald J. Overmars, Rob J. J. Beerkens, Jolanda J. C. Voermans, Shringar Rao, Henrieke A. B. Prins, Casper Rokx, Giuseppe Pantaleo, David A. M. C. van de Vijver, Tokameh Mahmoudi, Charles A. B. Boucher, Rob A. Gruters, Jeroen J. A. van Kampen

**Affiliations:** 1 Department of Viroscience, Erasmus University Medical Center, 3015 GD Rotterdam, The Netherlands; r.overmars@erasmusmc.nl (R.J.O.); r.beerkens@erasmusmc.nl (R.J.J.B.); j.voermans@erasmusmc.nl (J.J.C.V.); d.vandevijver@erasmusmc.nl (D.A.M.C.v.d.V.); c.boucher@erasmusmc.nl (C.A.B.B.); r.gruters@erasmusmc.nl (R.A.G.); j.vankampen@erasmusmc.nl (J.J.A.v.K.); 2 Service of Immunology and Allergy, Lausanne University Hospital, University of Lausanne, 1011 Lausanne, Switzerland; Francesco.Procopio@chuv.ch (F.A.P.); Giuseppe.Pantaleo@chuv.ch (G.P.); 3 Department of Biochemistry, Erasmus University Medical Center, 3015 GD Rotterdam, The Netherlands; s.rao@erasmusmc.nl (S.R.); t.mahmoudi@erasmusmc.nl (T.M.); 4 Department of Internal Medicine, Section of Infectious Diseases, Erasmus University Medical Center, 3015 GD Rotterdam, The Netherlands; h.prins@erasmusmc.nl (H.A.B.P.); c.rokx@erasmusmc.nl (C.R.)

**Keywords:** HIV-1 latency, reservoir quantification, inducible reservoirs, TILDA, assay performance

## Abstract

Substantial efforts to eliminate or reduce latent HIV-1 reservoirs are underway in clinical trials and have created a critical demand for sensitive, accurate, and reproducible tools to evaluate the efficacy of these strategies. Alternative reservoir quantification assays have been developed to circumvent limitations of the quantitative viral outgrowth assay. One such assay is *tat/rev* induced limiting dilution assay (TILDA), which measures the frequency of CD4+ T cells harboring inducible latent HIV-1 provirus. We modified pre-amplification reagents and conditions (TILDA v2.0) to improve assay execution and first internally validated assay performance using CD4+ T cells obtained from cART-suppressed HIV-1-infected individuals. Detection of *tat/rev* multiply spliced RNA was not altered by modifying pre-amplification conditions, confirming the robustness of the assay, and supporting the technique’s amenability to limited modifications to ensure better implementation for routine use in clinical studies of latent HIV-1 reservoirs. Furthermore, we cross-validated results of TILDA v2.0 and the original assay performed in two separate laboratories using samples from 15 HIV-1-infected individuals. TILDA and TILDA v2.0 showed a strong correlation (Lin’s Concordance Correlation Coefficient = 0.86). The low inter-laboratory variability between TILDAs performed at different institutes further supports use of TILDA for reservoir quantitation in multi-center interventional HIV-1 Cure trials.

## 1. Introduction

Novel experimental approaches designed to reduce or eliminate long-lived Human Immunodeficiency Virus type 1 (HIV-1) reservoirs are increasingly being tested in clinical trials, creating a demand for sensitive, accurate and reproducible assays to evaluate their potential effect on the size of the latent reservoir [1]. The quantitative viral outgrowth assay (QVOA) is used to measure the frequency of replication-competent proviruses that persist in a population of resting memory CD4+ T cells [2,3]. Although QVOA is considered the “gold standard”, this assay underestimates the size of the latent reservoir due to suboptimal induction of all replication-competent proviruses [4,5]. Simplified QVOA approaches, which are easier to perform, have been developed [2,6,7], but leukapheresis or large blood draws (up to 200 mL) are required to obtain sufficient amounts of input cells, due to the low frequency of latently infected cells (~1 replication-competent provirus per million resting CD4+ T cells). Furthermore, poor reproducibility and scalability limit its widespread use in clinical trials [7,8,9]. Alternatively, total HIV-1 DNA quantification using real-time or digital droplet PCR is straightforward and frequently used to determine HIV-1 reservoir size in clinical studies [10]. However, this technique largely overestimates reservoir size, because >98% of the proviruses detected are not replication-competent. Consequently, this assay is not suitable to study interventions that reduce the size of the replication-competent reservoir [5]. Alternative methods are under development to circumvent these limitations in estimating HIV-1 reservoir size. These include assays that quantify the amount of latently infected cells with inducible provirus (transcription-competent) or the number of cells capable of expressing viral proteins (translation-competent) [11,12,13,14,15,16]. However, these techniques also require large blood draws to get a sufficient amount of input cells (10–15 million CD4+ T cells) [12], which may not always be available in interventional clinical studies. A well-established inducible reservoir assay (*tat/rev* induced limiting dilution assay [TILDA]), which requires a minimum of 10–20 mL of blood, was developed to measure the frequency of CD4+ T cells expressing *tat/rev* multiply spliced RNA (msRNA), either constitutively or upon in vitro stimulation [17]. Although *tat/rev* msRNA expression does not exclusively translate into the production of infectious virions, the likelihood of quantifying defective proviruses with extensive internal deletions [5] is largely reduced. Indeed, TILDA holds great promise for application in clinical trials as it requires less input cells (<1 million CD4+ T cells) and can be completed within two days. The assay has a broad dynamic range (up to 3 logs), an inter-assay coefficient of variation (CV) of 21%, and a limit of detection (LOD) of 1.4 inducible reservoir cells per million CD4+ T cells [17]. However, in order for TILDA to be implemented in multi-center studies, it is essential to validate that this assay can be conducted within the unique infrastructure of each of the independent laboratories that preferentially use a fixed set of reagents, without compromising its reproducibility.

Here, we report that TILDA is amenable to limited modifications to facilitate its implementation for routine use in clinical studies of latent HIV-1 reservoirs. We also report the first evidence of low inter-laboratory variability between TILDAs performed at different institutes, thereby advocating its use for reservoir quantitation in multi-center interventional HIV-1 Cure trials.

## 2. Materials and Methods

### 2.1. Participants and Sample Collection

Samples from 36 HIV-1-infected individuals, >18 years of age on suppressive combination antiretroviral therapy (cART) with plasma HIV-1 RNA <50 copies/mL for at least six months were tested in this study. Except for one individual, all participants initiated cART during chronic HIV-1 infection. Peripheral blood mononuclear cells (PBMCs) were isolated from blood or leukapheresis samples by Ficoll density gradient centrifugation and were cryopreserved in liquid nitrogen. The characteristics of the study participants are detailed in Table S1.

### 2.2. Ethics Statement

Written informed consent was obtained from all participants. The use of samples for the study was approved by Erasmus MC Medical Ethics Committee (MEC-2005-227 [22 March 2006] and MEC-2016-148 [22 July 2016]).

### 2.3. Quantitative Plasma HIV-1 RNA Measurements

Plasma HIV-1 RNA was measured using a commercial HIV-1 RNA quantification assay (COBAS^®^ Ampliprep/COBAS^®^ Taqman^®^ HIV-1 Test v2.0, Roche Molecular Systems, Inc., Branchburg, NJ, USA) with a quantification limit of 20 copies/mL.

### 2.4. TILDA

TILDA was performed as initially described [17] or with several modifications (TILDA v2.0). Total CD4+ T cells were isolated from thawed PBMCs by negative magnetic selection using the EasySep Human CD4+ T cell Enrichment kit (STEMCELL Technologies, Vancouver, BC, Canada). CD4+ T cells were resuspended at 2 × 10^6^ cells/mL in complete RPMI-1640 (Lonza, Basel, Switzerland) supplemented with 10% Fetal Calf Serum (Sigma-Aldrich, Darmstadt, Germany) and 1% Penicillin/Streptomycin (Lonza, Basel Switzerland) and rested for 3 h at 37 °C and 5% CO_2_ prior to stimulation with 100 ng/mL phorbol 12-myristate 13-acetate (PMA) and 1 µg/mL ionomycin (both from Sigma-Aldrich, Darmstadt, Germany). After 12–14 h of stimulation, CD4+ T cells were washed in RPMI-1640, counted and serially diluted to 1.8 × 10^6^ cells/mL, 9 × 10^5^ cells/mL, 3 × 10^5^ cells/mL and 1 × 10^5^ cells/mL in RPMI-1640. From each dilution, 10 µL of the cell suspension was distributed to 22–24 wells of a 96-well plate containing 2 μL One-step RT-PCR enzyme (QIAGEN, Hilden, Germany), 10 μL 5× One-step RT-PCR buffer (QIAGEN, Hilden, Germany), 10 μL Triton-x100 (0.3%) (Sigma-Aldrich), 0.25 μL RNAsin (40 U/μL) (Promega, Leiden, The Netherlands), 2 μL dNTPs (10 mM each), 1 μL of forward and reverse primers [17] *tat* 1.4 and *rev* (both at 20 µM), and nuclease-free water to a final volume of 50 µL. A positive template control (in vitro-transcribed *tat/rev* RNA template) or ACH-2 cells (obtained through NIH AIDS reagent program, Germantown, MD, USA) and negative template control (nuclease free water) were added to two separate wells in each run. The primers (Eurogentec, Seraing, Liege, Belgium) and probe (Integrated DNA Technologies, Leuven, Belgium) used to detect multiply spliced *tat/rev* RNA are as described previously [17]. The one-step reverse transcription (RT)-PCR was run in a thermocycler (1000-series, Bio-Rad, Veneendal, The Netherlands) using the following thermocycling conditions: reverse transcription at 50 °C for 30 min, denaturation at 95 °C for 15 min, followed by 25 cycles of 95 °C for 30 s, 55 °C for 1 min and 72 °C for 2 min, and a final extension at 72 °C for 5 min. After one-step RT-PCR, 2 µL of the 1st PCR product was directly used as input for the *tat/rev* semi-nested real-time PCR reaction, which consisted of 5 µL 4× Taqman Fast Advanced Master Mix (Thermo Fisher Scientific, Landsmeer, The Netherlands), 0.4 µL of each primer (*tat* 2.0 and *rev*, both at 20 µM), 0.4 µL TILDA probe (5 µM), as published in [17], and nuclease-free water to a final reaction volume of 20 µL. The real-time PCR was performed in a LightCycler 480 Instrument II (Roche, Almere, The Netherlands) using the following conditions: 5 min at 50 °C (UNG step), 95 °C for 20 s, followed by 45 cycles of 95 °C for 3 s and 60 °C for 30 s. Positive wells at each dilution were counted, and the maximum likelihood method was used to determine the frequency of cells with inducible HIV-1 *tat/rev* msRNA using ELDA, a software application for extreme limiting dilution analysis [18] The major differences between the two TILDA methods are presented in Table 1.

### 2.5. Tat/rev RNA Standard and Cell Lines Used for Assay Validation

A gBlock containing a sequence of the *tat/rev* target region was synthesized by Integrated DNA Technologies (Leuven, Belgium) and in vitro RNA transcripts were generated using T7 Ribomax (Promega, Leiden, The Netherlands) and Turbo DNA-free kits according to manufacturer instructions (Ambion, Life Technologies, Landsmeer, The Netherlands). Serial dilutions of the DNase-treated synthetic *tat/rev* RNA were tested by qPCR with and without a reverse transcription step. The highest dilution that tested negative without RT and positive after RT was used as the final working stock. RNA concentration of this stock was determined using Qubit RNA HS Assay Kit (Invitrogen, Landsmeer, The Netherlands) and used to estimate copy numbers. ACH-2 cells (obtained from Thomas Folks through the AIDS reagent program, Division of AIDS, NIAID, NIH, Germantown, MD, USA) [19,20] and uninfected CD4+ T cells were maintained in complete RPMI-1640.

### 2.6. Statistical Analysis

Wilcoxon’s matched-pairs test was used to compare the median estimates generated from TILDA and TILDA v2.0. Lin’s Concordance Correlation analysis was used to compare the two methods and Bland-Altman plots to determine the limit of agreement. Statistical analyses were carried out using GraphPad Prism 5 Software (San Diego, CA, USA) and MedCalc Software (Ostend, Belgium). Two-tailed *p*-values of <0.05 were considered statistically significant.

## 3. Results

### 3.1. TILDA Is Amenable to Limited Modifications without Compromising Analytical and Clinical Sensitivity

**Figure 1:**
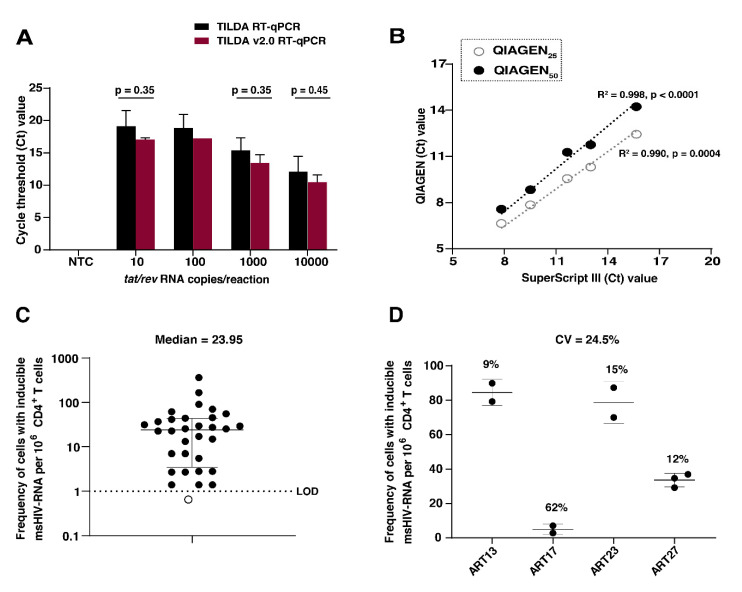
TILDA analytical and clinical sensitivity. (**A**) Detection of serial dilutions of a synthetic *tat/rev* RNA template in the absence of cells using the original TILDA RT-qPCR conditions compared to the TILDA v2.0 RT-qPCR conditions. Three wells per dilution were tested based on cycle threshold (Ct) values. NTC = Negative template control. A multiple paired *t*-test was used to compare mean Ct values. The error bars represent the SD of the mean of two independent runs. (**B**) Dilutions of 10–10,000 copies of *tat/rev* RNA were pre-amplified using different reaction volumes using QIAGEN (25 µL; open circles, and 50 µL; closed circles) or Superscript III Platinum one-step RT kits. Three wells per dilution were tested for positivity. A Pearson’s correlation test between the reference and two test kits is shown. Statistical significance is determined by *p* < 0.05. (**C**) Quantification of inducible HIV-1 reservoirs in clinical samples. Frequency of CD4+ T cells expressing *tat/rev* msRNA after in vitro stimulation with PMA/Ionomycin (*N* = 32 HIV-1-infected individuals on suppressive cART). Target was not detected for one sample, indicated using an open circle. The median and interquartile range is shown (the median frequency of cells with inducible *tat/rev* msRNA per million CD4+ T cells = 23.95). The dotted line represents the assay limit of detection (LOD). (**D**) The inter-assay coefficient of variation (CV) determined from TILDA measurements of four individuals. Mean CV= 24.5%. The other numbers represent the CV for each individual calculated from a minimum of two independent measurements. The error bars represent SD of the mean.

We modified TILDA as described in Table 1 to improve its implementation for routine use in clinical studies of latent HIV-1 reservoir analysis. To evaluate whether modifying the pre-amplification reagents and conditions would alter analytical sensitivity, we compared the capacity to detect serial dilutions of a synthetic *tat/rev* RNA template using reference (TILDA) or TILDA v2.0 RT-qPCR reagents and conditions. Regardless of the reagents used, there was no difference in detection of the *tat/rev* RNA standard at any dilution (*p* > 0.35) (Figure 1A). Additionally, when we compared target detection using different reaction volumes of the one-step RT-PCR kits (25 µL or 50 µL (QIAGEN) and 11 µL (Superscript III Platinum), there was a strong correlation (R^2^ ≥ 0.99) between the cycle threshold (Ct) values obtained for both kits (Figure 1B). We opted for 50 µL as the final reaction volume for the one-step RT-PCR, which permits larger sample input volumes, minimizing pipetting imprecisions. To evaluate TILDA v2.0 performance in clinical samples, we isolated CD4+ T cells from PBMC obtained from 36 HIV-1-infected individuals, suppressed on cART for more than six months. *Tat/rev* msRNA was detected in 31 out of 32 (96.9%) HIV-1 subtype B samples tested. Samples from individuals infected with undetermined or non-B HIV-1 subtype (*n* = 4) were excluded from further analysis, as *tat/rev* msRNA is less efficiently detected due to primer and probe specificity for subtype B viruses. However, two of these samples had detectable levels of *tat/rev* msRNA. The frequencies of CD4+ T cells expressing *tat/rev* msRNA from 32 individuals with subtype B infection are shown in Figure 1C. The median frequency of cells expressing *tat/rev* msRNA was 23.95 cells per million CD4+ T cells (Range: 1.4–362). The inter-assay coefficient of variation (CV) for TILDA v2.0 was 24.5% (Range: 9–62%) (Figure 1D), which is comparable to that of the original assay (CV = 21%) [17]. We measured cell viability of two independent replicates for each sample after stimulation with PMA and Ionomycin and observed no apparent correlation between the CV and difference in cell viability. Overall, modifications of the RT-qPCR did not alter the detection of *tat/rev* msRNA and subsequent quantification of the inducible reservoir in clinical samples, demonstrating that this technique is amenable to modifications to facilitate personalized application in clinical settings.

### 3.2. TILDA Shows High Inter-Laboratory Reproducibility

**Figure 2:**
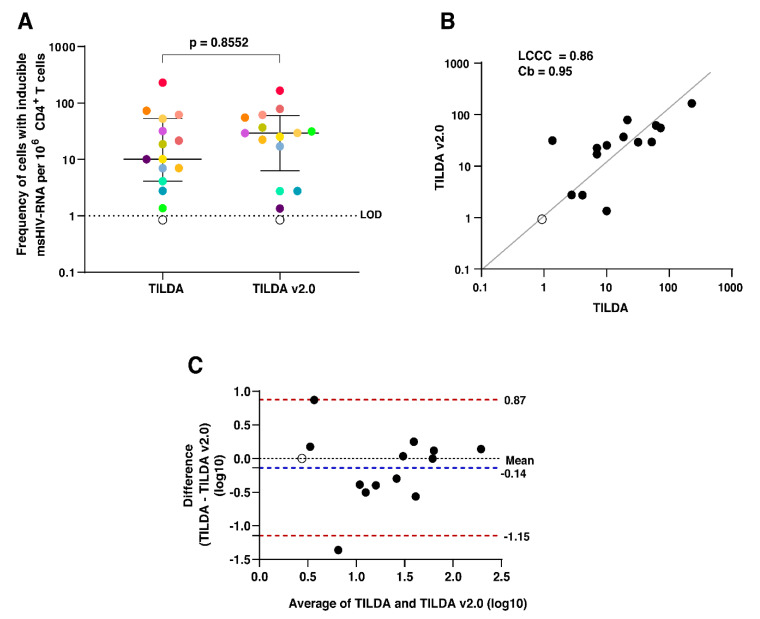
TILDA inter-laboratory reproducibility. (**A**) The frequency of CD4+ T cells expressing *tat/rev* msRNA after in vitro stimulation with PMA/Ionomycin in a subset of individuals (*N* = 15), quantified by TILDA and TILDA v2.0. Each individual is represented by a different colored dot. The open circles are below the LOD. Wilcoxon’s matched-pairs test was used to compare the medians. Statistical significance is determined by *p* < 0.05. (**B**) Lin’s Concordance Correlation Coefficient (LCCC) plot. The black dots represent inducible reservoir size quantified by TILDA and TILDA v2.0. The open circle is below the LOD. The grey diagonal line represents the best-fit line. Coefficient of bias (Cb) is a measure of accuracy. (**C**) Bland–Altman plot using log-transformed inducible reservoir size measurements by the two assays. The open circle is below the LOD. The blue dotted line represents the mean bias, and the red dotted lines represent the 95% limits of agreement.

In an inter-laboratory assessment of TILDA and TILDA v2.0, cryopreserved PBMCs were shipped from Erasmus MC in Rotterdam to CHUV, Department of Immunology and Allergy in Lausanne, to compare the estimated frequencies of CD4+ T cells expressing *tat/rev* msRNA in a subset of samples (*n* = 15). A different operator performed TILDA according to the initially described protocol [17]. As shown in Figure 2A, there was no significant difference in the median estimate and IQR for the two versions (*p* = 0.8552), which correlated well (Lin’s Concordance Correlation Coefficient = 0.86, Coefficient of bias = 0.95) (Figure 2B). A Bland–Altman plot revealed a strong agreement between the two versions of the assay (mean difference = −0.14 log10 with 86.6% of the data points falling within the 95% Limits of agreement (LOA) −1.15 to 0.87 log10) (Figure 2C). These data support the cross-comparison of TILDA results generated by different methods performed in different laboratory settings.

## 4. Discussion

TILDA [17] is a widely adopted method used to measure latent HIV-1 reservoirs in different groups of people living with HIV-1. Due to its low sample requirement and short turn-around time compared to QVOA, TILDA is an appropriate alternative for quantifying the replication-competent HIV-1 reservoir and allows easier longitudinal sample analysis. In this study, we modified TILDA to increase its amenability for routine use in clinical studies of the latent HIV-1 reservoir and assessed the inter-laboratory reproducibility of the initial and modified assay (TILDA v2.0). Our results provide additional evidence that TILDA is amenable to modifications without compromising assay performance, facilitating its routine implementation in diagnostic/laboratory settings that have already optimized/validated their protocols using a fixed set of RT-qPCR reagents. The robustness of TILDA thus enables the recapture of available laboratory resources, resulting in the reinvestment of surplus reagents and saving laboratory costs by purchasing reagents in larger volumes, which in the long run may improve internal cost-effectiveness. Several others have also reported the optimization and further development of TILDA either through the modification of primers to detect *tat/rev* msRNA expressed from non-B HIV-1 subtypes, isolating RNA prior to RT-qPCR to improve assay sensitivity or by enhancing the inducibility of *tat/rev* msRNA in CD4+ T cells from infants through the addition of phytohemagglutinin and a longer duration of stimulation (18 instead of 12 h) [21,22,23,24,25,26]. Our method increases the reaction volumes of alternative PCR reagents used to pre-amplify *tat/rev* msRNA to permit larger sample volumes (10 µL compared to 1 µL in the initial TILDA protocol), which reduces pipetting imprecisions. Moreover, cells may be dispensed using multi-step pipettes, reducing operating time, which is critical because rapid processing of the sample is essential for minimizing *tat/rev* msRNA degradation after stimulation and to avoid underestimating the reservoir size. Favorably, pre-amplifying *tat/rev* msRNA in a 50 µL reaction volume compared to 11 µL in the initial TILDA protocol eliminates the need to dilute the PCR products prior to target detection by real-time PCR. This modification not only reduces several pipetting steps in the workflow, but also minimizes the risk of cross-contamination when diluting the first amplification products, which is essential because false positives would result in the overestimation of reservoir size. Therefore, we advocate modifications such as those in TILDA v2.0 to improve the technical operation of the assay and minimize errors in quantifying inducible reservoirs. While the inter-assay variation of TILDA was determined in this study and in [17], there is no data yet on the assay’s inter-laboratory reproducibility. Using 15 clinical samples, we report a strong agreement between the two TILDA methods with a Lin’s Concordance Correlation Coefficient (LCCC) of 0.86. In contrast, QVOA, considered the reference assay for replication-competent reservoir quantification, is more variable [7,8,9]. The variation observed in QVOA measurements is attributable to several factors related to the complexity of the assay such as the number of input cells and replicates, different potencies of activating compounds, the duration of stimulation, different feeder cell type used to amplify reactivated virus and different methods of readout [6,7,9,27,28,29]. Furthermore, applying Poisson distribution to detect rare cells harboring replication-competent provirus is a major unavoidable source of variation and dictates the input number of cells required [7,9]. With the development of newer techniques to quantify the reservoir, it is imperative that each reservoir quantification method is extensively evaluated for assay performance criteria such as specificity, sensitivity, and reproducibility within and between laboratories, alongside scalability to assess the validity for their application in HIV-1 cure trials. Moreover, given the small changes in reservoir size currently attainable with the available clinical interventions, the methods developed should accurately and precisely measure reservoir dynamics in a reproducible manner in multi-center clinical studies and longitudinal patient samples. Our data on negligible inter-laboratory variability of TILDA despite limited modifications to the protocol to improve technical operation further advocate the application of TILDA for reservoir quantification and cross-comparison of results from the initial assay or modified versions performed in multi-center HIV-1 cure clinical trials. Further research is warranted to investigate TILDA’s suitability for measuring reservoir dynamics in reservoir-reduction clinical intervention studies.

## Figures and Tables

**Table 1:** Major differences in RT-qPCR conditions in the two versions of TILDA.

Characteristic	TILDA	TILDA v2.0
**Input volume of cells per reaction ^1^**	1 µL	10 µL
**One-step RT-PCR reagents**	Superscript III Platinum Taq (Life Technologies) & Buffer, RNase inhibitor (Life Technologies), Tris-EDTA (TE) buffer	HotStarTaq DNA Polymerase, Sensiscript and Omniscript Reverse Transcriptases & Buffer (QIAGEN), RNasin Ribonuclease Inhibitor (Promega), 0.1% solution of Triton X-100
**Final reaction volume**	11 µL	50 µL
**Cycling conditions**	50 °C for 15 min, 95 °C for 2 min, 24 cycles of amplification (95 °C 15 s, 60 °C 4 min)	50 °C for 30 min, 95 °C for 15 min, 25 cycles of amplification (95 °C 30 s, 55 °C 1 min, 72 °C 2 min) final extension at 72 °C for 5 min
**Input volume of pre-amplified product per reaction**	1 µL of 1:5 dilution	2 µL
**Real-time PCR reagent**	LightCycler 480 Probe Master buffer (Roche Applied Sciences)	Custom TaqMan Fast Advanced Master Mix (Thermo Fisher Scientific)
**Final reaction volume**	10 µL	20 µL
**Cycling conditions**	95 °C for 10 min, 45 cycles of 95 °C 10 s, 60 °C 30 s, 72 °C 1 s and a cooling step at 40 °C for 30 s	50 °C for 5 min (UNG step), 95 °C for 20 s, 45 cycles of 95 °C for 3 s and 60 °C for 30 s

^1^ Cells are serially diluted and then distributed in replicates of 18,000, 9000, 3000 and 1000 per well with the indicated volume. Primers and probe as published [17].

## Supplementary Materials

The following are available online at https://www.mdpi.com/1999-4915/12/9/973/s1, Table S1: Characteristics of study participants.
