# Supplementary material for: Inter-Laboratory Reproducibility of Inducible HIV-1 Reservoir Quantification by TILDA
Source: Viruses. 2020 Sep 2;12(9):973. doi: 10.3390/v12090973 (PMC7552071; doi:10.3390/v12090973)
Supplement: Supplementary file 1 [file viruses-12-00973-s001.pdf]

Table S1. Characteristics of study participants

ID	Age (years)	CD4 nadir (cells/mm³)	HIV-1 subtype	Pre-cART plasma HIV-1 RNA (log10 copies/mL)	Time on cART (years)	CD4+ T cell count (cells/mm³)	TILDA v2.0 (Upper and lower 95% CI)
ART02	51	400	B	>5.0	6.9	790	2.8 (0.7, 11)
ART03	50	600	B	4.75	5.6	1100	27.3 (16.9, 44.1)
ART04	26	750	B	5.64	4.0	1020	13.1 (6.8, 25.2)
ART05	29	390	B	4.78	1.6	540	14.8 (7.9, 27.5)
ART06	64	30	B	5.50	2.2	390	362 (253.7, 516.2)
ART08	57	420	B	7.21	2.1	1120	5.5 (2.1, 14.8)
ART09	29	520	B	5.23	1.8	520	43.7 (26.9, 71)
ART10	52	420	B	4.74	1.3	790	25.5 (15, 43.2)
ART11	33	980	B	5.27	1.5	790	2.7 (0.7, 10.9)
ART13	41	220	B	>5.0	7.2	710	90 (65.1, 124.3)
ART14	23	470	B	4.75	1.4	1360	2.7 (0.7, 10.9)
ART15	50	270	B	3.95	5.5	580	1.4 (0.2, 9.6)
ART16	29	390	B	4.17	2.0	700	TND
ART17	24	430	B	4.85	1.3	670	7.0 (2.9, 16.9)
ART19	41	660	B	4.86	0.8	660	7.0 (2.9, 16.9)
ART20	40	230	B	5.17	6.4	730	2.8 (0.7, 11.1)
ART21	57	350	B	5.14	1.4	840	41.2 (27.2, 62.4)
ART22	25	1060	B	4.20	2.6	1590	22.4 (12, 41.7)
ART23	44	530	B	5.65	4.3	960	70 (49.4, 99.4)
ART24	45	220	B	4.81	8.5	490	22.7 (12.6, 41.1)
ART25	37	340	B	4.29	2.7	810	1.4 (0.2, 9.6)
ART26	36	440	B	4.57	9.9	960	1.4 (0.2, 9.6)
ART27	41	370	B	5.31	6.2	570	29.2 (18.4, 46.5)
ART28	56	400	B	4.73	10.5	590	31.3 (19.9, 49.2)
ART29	42	650	B	4.87	10.6	730	61.5 (41.6, 91)
ART30	48	450	B	5.01	9.7	940	55.3 (38.3, 79.7)
ART31	42	340	B	5.03	8.8	510	29.4 (18.7, 46.3)
ART32	45	310	B	5.01	7.4	580	36.8 (24.1, 56.1)
ART33	37	300	B	4.85	10.5	680	45.3 (25.5, 79.5)
ART34	42	290	B	4.01	2.9	580	17 (9.4, 30.7)
ART35	56	350	B	NA	11.7	700	25.2 (15.4, 41.3)
ART36	50	350	B	5.15	3.5	650	165.7 (121.7, 225.2)
Non-B HIV-1 subtype							
ART01	68	310	ND	5.50	13.1	770	TD
ART07	54	250	AG	4.92	1.4	370	TND
ART12	36	NA	ND	NA	3.0	410	TND
ART18	46	141	G	6.36	2.2	410	TD

TD: target detected, TND: target not detected, 95% CI: 95% Confidence interval, NA: not available, ND: not determined
